# Different subcellular localizations and functions of Arabidopsis myosin VIII

**DOI:** 10.1186/1471-2229-8-3

**Published:** 2008-01-08

**Authors:** Lior Golomb, Mohamad Abu-Abied, Eduard Belausov, Einat Sadot

**Affiliations:** 1 The Institute of Plant Sciences, The Volcani Center, Bet-Dagan 50250, Israel

## Abstract

**Background:**

Myosins are actin-activated ATPases that use energy to generate force and move along actin filaments, dragging with their tails different cargos. Plant myosins belong to the group of unconventional myosins and Arabidopsis myosin VIII gene family contains four members: ATM1, ATM2, myosin VIIIA and myosin VIIIB.

**Results:**

In transgenic plants expressing GFP fusions with ATM1 (IQ-tail truncation, lacking the head domain), fluorescence was differentially distributed: while in epidermis cells at the root cap GFP-ATM1 equally distributed all over the cell, in epidermal cells right above this region it accumulated in dots. Further up, in cells of the elongation zone, GFP-ATM1 was preferentially positioned at the sides of transversal cell walls. Interestingly, the punctate pattern was insensitive to brefeldin A (BFA) while in some cells closer to the root cap, ATM1 was found in BFA bodies. With the use of different markers and transient expression in *Nicotiana benthamiana *leaves, it was found that myosin VIII co-localized to the plasmodesmata and ER, colocalized with internalized FM4-64, and partially overlapped with the endosomal markers ARA6, and rarely with ARA7 and FYVE. Motility of ARA6 labeled organelles was inhibited whenever associated with truncated ATM1 but motility of FYVE labeled organelles was inhibited only when associated with large excess of ATM1. Furthermore, GFP-ATM1 and RFP-ATM2 (IQ-tail domain) co-localized to the same spots on the plasma membrane, indicating a specific composition at these sites for myosin binding.

**Conclusion:**

Taken together, our data suggest that myosin VIII functions differently in different root cells and can be involved in different steps of endocytosis, BFA-sensitive and insensitive pathways, ER tethering and plasmodesmatal activity.

## Background

The Arabidopsis myosin gene family contains 17 members. The myosin XI group, which is related to unconventional myosin V [1,2], includes 13 members while the myosin VIII group, which is related to unconventional myosin V [2] but also to myosin VI [1], includes four members. Both myosin groups VIII and XI are specific to plants [3]. Typically, the Arabidopsis myosins contain a conserved motor domain, a number of IQ domains for light-chain binding, a coiled-coil domain that is predicted to facilitate their dimerization and a specific tail to bind the cargo [3]. Plant myosins are generally implicated in cytoplasmic streaming [4], organelle movement [5-7], cytokinesis [8-10], plasmodesmatal functioning [10,11], and endocytosis [10,12,13].

Myosin VIII (ATM1) was the first plant myosin to be identified and sequenced [14]. A specific antibody raised against a peptide corresponding to its unique tail was used to show, in both immunofluorescence and electron microscopy studies, that ATM1 was localized to plant-specific structures such as the plasmodesmata and plasma membrane of newly formed cell walls in root cells of maize and Arabidopsis [11,15]. ATM1 has also been found in pit fields in the inner cortex cells of maize root apices, where it was predicted to be involved in fluid-phase endocytosis [12]. In a more recent work, the same antibody was used to show that in cells of maize root caps, ATM1 localized around the nuclei but relocated to amyloplasts and to plasmodesmata following 5 min and 90 min of osmotic stimulus, respectively [16]. The latter suggests acto-myosin involvement in root osmo-sensing [16]. When ATM1 was fused to GFP, its fluorescence was concentrated mostly at the developing cell plate in BY2 cells [17]. The four members of myosin VIII are ATM1, ATM2, myosin VIIIA and myosin VIIIB. While ATM1 is more similar to VIIIA, ATM2 is related to VIIIB both in sequence and expression pattern.

In this work, we followed myosin VIII using GFP fusions. We show that in transgenic plants expressing GFP-ATM1(IQ-tail) fluorescence is differentially localized in different root cells. Sensitivity to BFA also differed between root cells. When transiently expressed in *N. benthamiana*, GFP-ATM1(IQ-tail) was found in pit fields accumulating callose, and co-localized with the ER. ATM1 also co-localized with internalized FM4-64 and partially co-localized with the endosomal markers ARA6, and rarely with ARA7 and FYVE. Furthermore, truncated ATM1 inhibited the motility of associated ARA6 labeled organelles but could be found on motile FYVE labeled organelles. Only large excess of ATM1 associated with FYVE labeled organelles arrested their motility. Taken together our data suggest that myosin VIII differentially functions in different cells and can be involved in different steps of endocytosis, BFA sensitive and insensitive pathways, ER tethering and in plasmodesmatal activity.

## Results

### Transgenic plants expressing GFP-ATM1(IQ-tail)

Transgenic Arabidopsis plants expressing GFP-ATM1(IQ-tail) were generated. Among tens of seedlings resistant to kanamycin, only one seedling expressed detectable levels of GFP-ATM1(IQ-tail), suggesting some cytotoxicity of the mutant molecule. GFP-ATM1(IQ-tail) was expressed and passed on to subsequent generations but no significant phenotypes or changes in cell morphology relative to wild-type (wt) plants were observed. Interestingly, fluorescence of GFP-ATM1 (IQ-tail) was visible in the roots but almost undetectable in the shoots and leaves.

**Figure 1:**
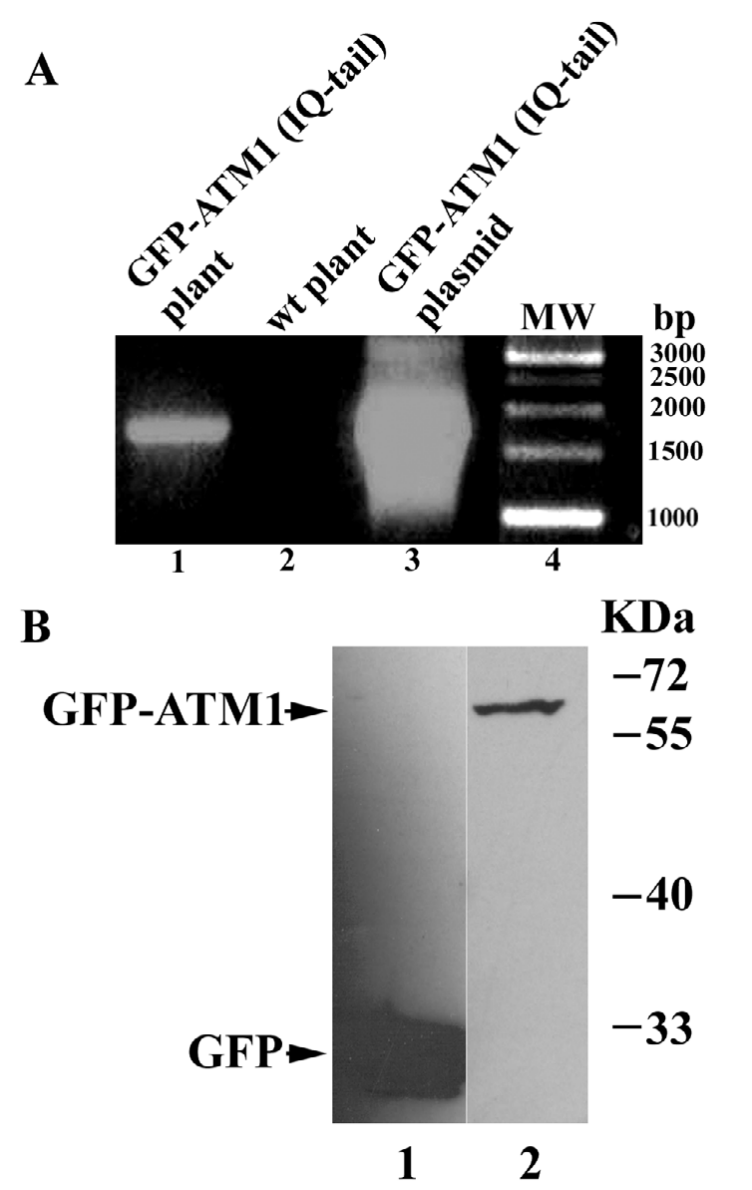
**Verification of GFP-ATM1(IQ-tail) expression in transgenic plants**. A. PCR was performed using a forward primer corresponding to the 5' end of GFP starting from the ATG and a reverse primer corresponding to the 3' end of ATM1 including its stop codon. The size of the expected fragment was 1734 bp. The template DNA was as follows: Lane 1. DNA from transgenic plants expressing GFP-ATM1(IQ-tail). Lane 2. DNA from wt plants. Lane 3. DNA from the plasmid used to generate the transgenic plants. Lane 4. Molecular weight markers. B. Western blot analysis showing sizes and levels of the expressed transgenes: Lane 1. GFP alone. Lane 2. GFP-ATM1(IQ-tail). Detection was performed with anti-GFP antibody.

**Figure 2:**
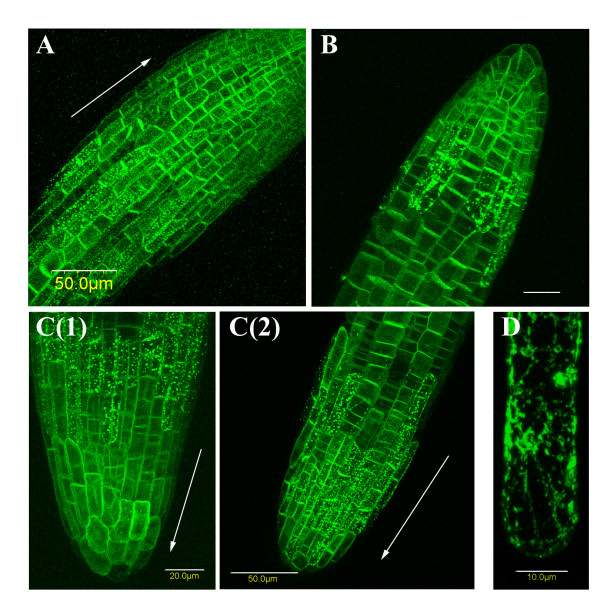
**Differential localization of GFP-ATM1(IQ-tail) in root cells**. Serial optic sections (30–70, 0.8 μm apart) of roots were acquired by confocal microscopy. A. Root of 5-day-old seedling. Scale bar: 50 μm. B. Lateral root of 20-day-old seedling. Scale bar: 20 μm. C(1) and C(2). Two images of the same 20-day-old seedling root. C(1) shows the root cap, scale bar: 20 μm, and C(2) shows the upper part. Scale bar: 50 μm. A similar pattern of GFP-ATM1 localization is seen in all roots: diffuse at the root cap, then dots, then more polarized organization along the transverse sides. D. GFP-ATM1 in root hair, scale bar: 10 μm. Arrows show the direction of the root caps.

To verify that the chimera is expressed properly, PCR was performed on DNA prepared from the transgenic plants and from wt plants as a control, and compared to PCR of DNA from the plasmid encoding the chimera used to generate the transgenic plants. Figure 1A shows that the full length of the expected fragment (1734 bp) was detected in the PCR using DNA from transgenic plants similar to the fragment obtained from plasmid DNA. Control plants were negative. Western blot analysis confirmed the expression of the full-length chimera (~57 kDa) and showed that the level of expression of the GFP-ATM1 chimera in transgenic plants is very low compared to the expression of GFP alone in control plants (Figure 1B). Because of the lack of good anti ATM1 antibodies in our hands, no comparison to endogenous protein's level of expression was done. Confocal-microscope images of roots of the transgenic plants revealed that the chimera GFP-ATM1(IQ-tail) is differentially localized in different cells. While in epidermis cells at the root cap fluorescence was equally distributed all over the cell, in epidermal cells right above this region fluorescence accumulated in dots or aggregates of 850 ± 150 nm in diameter. Further up, in cells of the elongation zone, GFP-ATM1(IQ-tail) was preferentially positioned at transverse cell walls (Figure 2A, B and 2C). This pattern of GFP-ATM1(IQ-tail) subcellular distribution was found in roots of 5-day-old (Figure 2A) and 20-day-old (Figure 2B and 2C) seedlings, including the lateral roots (Figure 2B) of 20-day-old seedlings. When a three dimension image of figure 2B was constructed and rotated around its axis, it could be distinguished that the dots were scattered around the cells making it difficult to distinguish between membrane and cytoplasmic specific localization (Additional file 1). In root hairs, GFP-ATM1(IQ-tail) also formed a pattern of dots (Figure 2D).

### Differential sensitivity of ATM1 to BFA in different root cells

**Figure 3:**
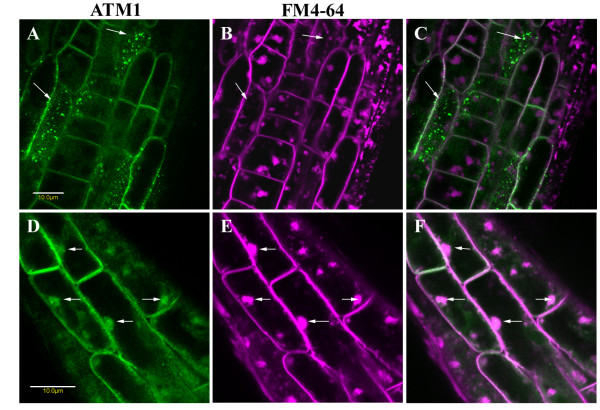
**Association of ATM1 with BFA bodies in specific cells**. Seedlings were treated with BFA and FM4-64 and image acquisition was performed with a confocal microscope. A-B. Cells with GFP-ATM1(IQ-tail) organized in dots (arrows). A. GFP-ATM1. B. BFA bodies formed in these cells, shown by FM4-64. Note that the dotted pattern is not disrupted by the treatment (arrows). C. Overlay of A and B. Scale bar 10 μm. D-F Showing cells near the root cap where ATM1 is found in BFA bodies. D. GFP-ATM1, E. BFA bodies stained by FM4-64, F. overlay of D and E. Scale bar 10 μm. All images in this figure are composed of one optic section.

Unlike in several other species where BFA treatment leads to redistribution of Golgi proteins to the ER [18], in Arabidopsis root cells, BFA leads to the formation of BFA-bodies that are derived from endosomal membranes and accumulate endocytosed markers [19-22]. Thus it has been shown that the BFA-sensitive process in Arabidopsis is endosomal trafficking [19,20,22-24]. Since myosin VIII has been implicated in endocytosis [10,12,13] we addressed the question of whether BFA treatment would affect the specific subcellular organization of GFP-ATM1(IQ-tail) in the different root cells and whether ATM1 would be found in the BFA bodies. Figure 3A, B and 3C shows that BFA treatment did not disrupt the punctate pattern of ATM1 and no ATM1 was found in the BFA bodies formed in these particular cells; suggesting that here, ATM1's function is BFA-independent. In contrast, in cells closer to the root cap where ATM1 was equally distributed all over the cell before BFA treatment, after BFA treatment it was found in BFA bodies (Figure 3D, E and 3F). This suggests that in these cells, ATM1 is BFA-sensitive and might be involved in endosomal trafficking at specific developmental stages. For comparison and control, transgenic seedling expressing GFP alone were treated with BFA and FM4-64. In these plants GFP was diffused all over the cytoplasm of root cells where BFA bodied were detected (Additional file 2). To check whether ATM1 is involved in different steps of endocytosis, we used a variety of different endosomal markers.

### Sub-cellular localization of ATM1 transiently expressed in *N. benthamiana*

**Figure 4:**
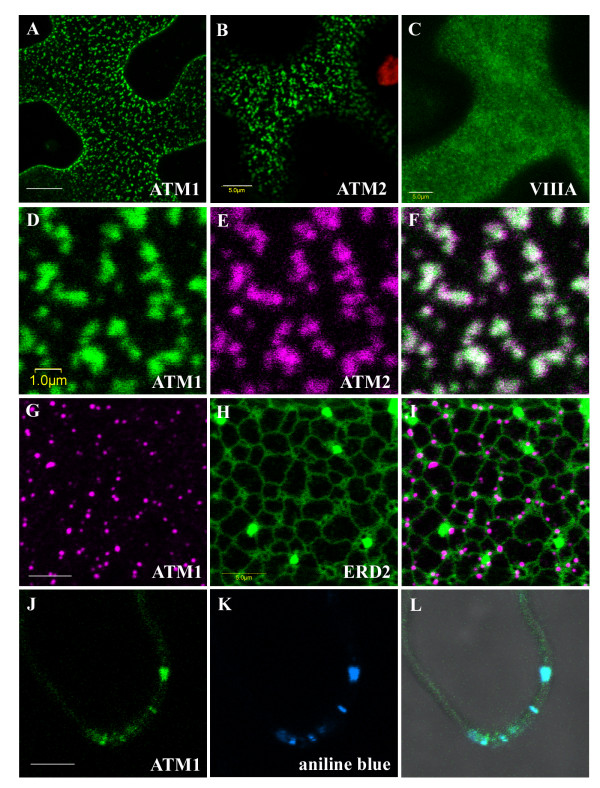
**Subcellular localization of myosin VIII in abaxial leaf epidermis cells of *N. benthamiana***. Fluorescent chimeras were co-expressed by Agrobacterium infiltration. A. GFP-ATM1(IQ-tail). Scale bar 10 μm, 15 optic sections, 0.5 μm apart. B. GFP-ATM2 (IQ-tail) Scale bar 5 μm, 1 optic section. C. GFP-myosin VIIIA (IQ-tail). Scale bar 5 μm, 8 optic sections, 0.5 μm apart. D. GFP-ATM1(IQ-tail). E. RFP-ATM2(IQ-tail). F. Overlay of D and E. Scale bar 1 μm, 1 optic section. G. RFP-ATM1(IQ-tail). H. ERD2-GFP. I. Overlay of G and H. Scale bar 5 μm, 1 optic section. J. GFP-ATM1(IQ-tail). K. Aniline blue labeling of callose accumulated in pit fields. L. Overlay of J and K. Scale bar 5 μm, 1 optic section.

**Figure 5:**
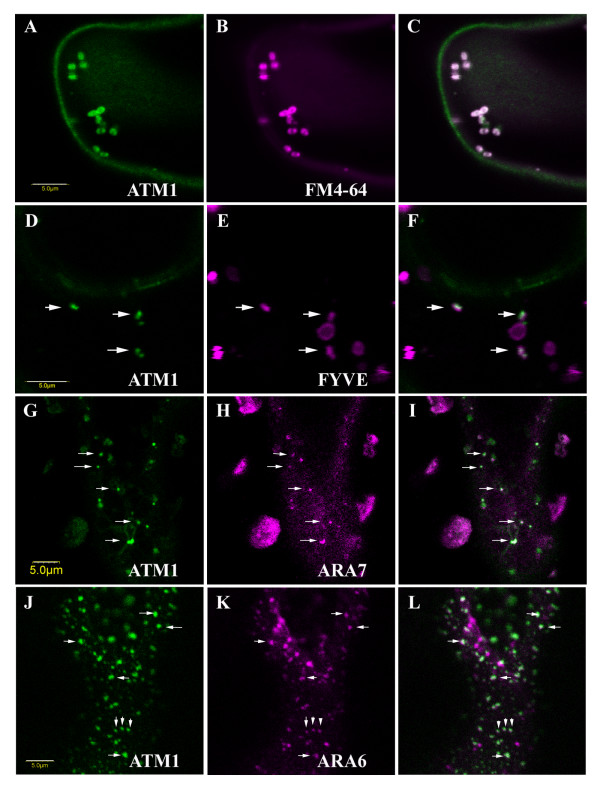
**ATM1 co-localizes with internalized FM4-64 and with endosomal markers in abaxial leaf epidermis cells of *N. benthamiana***. Fluorescent chimeras were co-expressed by Agrobacterium infiltration. A. GFP-ATM1(IQ-tail) (dots of 980 ± 145 nm in diameter). B. FM4-64. C. Overlay of A and B (1 optic section). D. GFP-ATM1(IQ-tail) (dots of 630 ± 60 nm). E. FYVE-DsRED. F. Overlay of D and E (1 optic section). G. RFP-ATM1(IQ-tail) (dots of 300 ± 100, colored green for ease of demonstration). H. GFP-ARA7 (colored magenta for ease of demonstration). I. Overlay of G and H (1 optic section). J. RFP-ATM1(IQ-tail) (dots of 570 ± 75 nm, colored green for ease of demonstration). K. ARA6-GFP (colored magenta for ease of demonstration). L. Overlay of J. and K (1 optic section). Arrows show co-localization. Scale bars 5 μm. The microscope focus in A-I was in the cytoplasm while the focus in J-L was on the plasma membrane.

When fluorescent chimeras (GFP or RFP) of ATM1(IQ-tail) were transiently expressed in *N. benthamiana *leaves using Agrobacterium infiltration, fluorescence accumulated as dots (or aggregates of dots) on the plasma membrane of abaxial leaf epidermal cells (Figure 4A). To verify whether this is a general pattern of localization for myosin VIII members, we checked ATM2, myosin VIIIA and myosin VIIIB. ATM2 and ATM1 gave a similar pattern of dots (590 ± 180 nm in diameter) while myosin VIIIA formed smaller dots (330 ± 50 nm in diameter) (Figure 4B and 4C). Myosin VIIIB was very similar to myosin VIIIA (not shown). The vast majority of ATM1 fluorescent dots were stationary but rarely, less than 1% of the dots were motile (Additional file 3). The family members of myosin VIII exhibit different expression patterns as shown by genevestigator analysis (Additional file 4) [25]. While ATM1 and myosin VIIIA are similarly expressed in most organs, ATM2 and myosin VIIIB are more highly expressed in pollen and to a lesser extent in the stamen and root hairs (Additional file 4). It was thus interesting to determine whether the dots formed by ATM1 and ATM2 overlap in *N. benthamiana *leaves. GFP-ATM1 and RFP-ATM2 were therefore co-expressed in these leaves, and both localized to the same specific spots on the plasma membrane (Figure 4D–F). This suggests the existence of specific foci at the plasma membrane that are able to bind both myosins. Interestingly, when the ATM1 fluorescent chimera was co-expressed with an ER marker, ERD2-GFP [26], the punctate labeling pattern of ATM1 correlated with the ER (Figure 4G–I). The same picture was observed with ATM2 (not shown). Using aniline blue to stain callose [27], ATM1 was shown to accumulate in plasmodesmata-enriched pit fields (Figure 4J–L). To extend our view on the possible involvement of ATM1 in endocytosis the membrane dye FM4-64 was used to follow membrane internalization. ATM1 was found not only at the plasma membrane, but also in the cytoplasm where it co-localized with internalized FM4-64 (Figure 5A–C). In addition, fluorescent chimeras of ATM1 were co-expressed with the endosomal markers ARA6-GFP and GFP-ARA7, Rab5 orthologs from Arabidopsis [28], and DsRed-FYVE. The FYVE domain is a conserved protein motif characterized by its ability to bind with high affinity and specificity to phosphatidylinositol 3-phosphate (Pi(3)P), a phosphoinositide that is highly enriched in early endosomes [29] and has been shown to co-localize with ARA7 and with internalized FM4-64 in plants [30]. It was found that while FYVE and ARA7 labeled organelles were in the cytoplasm, in less than 1% of the labeled organelles, colocalization with ATM1 was observed (Figure 5D–F, G–I). Generally, the motility of FYVE and ARA7 labeled organelles was not affected by the presence of truncated ATM1 in the same cell, probably because most of them were not colocalized (Additional file 5). However, occasionally both motile organelles co-labeled with FYVE and ATM1 (Additional file 6), and motionless FYVE labeled bodies surrounded by excess of GFP-ATM1 could be detected (Additional file 7). About 80–90% of ARA6 labeled organelles colocalized with ATM1 at or close to the plasma membrane (Figure 5J–L). Importantly, while ARA6 labeled organelles were highly motile in the absence of GFP-ATM1 (additional file 8), in the presence of ATM1, all co-labeled organelles, became motionless and only those free from ATM1 remained motile (Additional file 9). The above suggests a major role for ATM1 in the function of ARA6 labeled endosomes and a minor role in the function of ARA7/FYVE labeled endosomes [31].

## Discussion

Here, we studied transgenic plants expressing a chimera of GFP fused to a truncated myosin VIII – the IQ-tail domain of ATM1. This mutant molecule, lacking the head, motor domain that bind actin but containing the neck and tail domains, is expected to bind to its cellular targets and function as dominant negative by blocking wt myosin binding. Indeed, among many seedlings expressing the gene for antibiotic resistance, only one was found expressing the fluorescent chimera of myosin, at very low levels. This might be the result of a cytotoxic effect of the mutant. The fact that no detectable phenotypes were observed in the plants expressing the mutant myosin was a surprise, but might be explained by the low level of expression. In this regard, it should be mentioned that when other truncated myosins were expressed in plant cells, no significant inhibition of organellar movement was observed [32] suggesting redundancy in myosin's function. In the transgenic plants, we show differential localization and function of ATM1 in root cells. Similar differential localization was shown for myosin VIII in maize roots stained with anti myosin VIII antibody where it was found to be distributed diffusely in root cap cells but appeared as fine spots in the distal part of the apical meristem, in cells of the inner cortex, and in the distal part of the elongation region [11].

In view of its differential sensitivity to BFA in different root cells and its co-localization with different endosomal markers, we propose that ATM1 participates in various stages of endosomes biogenesis and function. We confirm previous data that myosin VIII resides at the plasma membrane and is enriched in plasmodesmata [11,12,15,33]. In addition, we confirm a previous conclusion that myosin VIII is involved in endocytosis [13] and participates in tethering cortical elements of the ER to the plasma membrane [10].

### ATM1 at the plasma membrane and endosomes

Based on their findings, Dieter Volkmann and co-workers concluded that myosin VIII may be less important for intracellular motility and more involved in the anchoring of actin filaments at cell peripheries [15]. They predicted another possible role for myosin VIII in forming a structural support for the cortical ER elements tightly underlying the plasma membrane, both outside and inside the plasmodesmata [10]. They also suggested that myosin VIII might drive invagination of the plasma membrane during fluid-phase endocytosis [12].

While ATM1 is more or less equally expressed in all plant organs, ATM2 show specificity to male reproductive organs (Additional figure 2). Other gene families of cytoskeletal proteins show differential expression in vegetative and reproductive tissues, such as actin [34], profilin [35,36] and myosin XI (genevestigator expression profiles [25]). We provide evidence here that both ATM1 and ATM2 co-localize at specific sites on the plasma membrane when transiently expressed in *N. benthamiana *leaf. This is the first time that two plant myosins have been shown to co-localize at the same spots on the plasma membrane, suggesting a unique composition of the plasma membrane at these sites. The myosin VIII-labeled spots on the plasma membrane might be sites where the cortical actin fibers are linked to the plasma membrane such as "focal contacts" [10,37,38], sites of endocytosis [31], sterol-enriched complexes [24] or something else that we do not yet know about. Since our myosin constructs do not contain the head domain which is the actin-binding domain, we could not use them to address the question of actin binding to the plasma membrane.

In addition, we show co-localization of ATM1 (Figure 5G–I) with the ER in *N. benthamiana *leaves. When BDM (2,3-Butanedione 2-monoxime) which is a general inhibitor of myosin ATPases of eukaryotic cells, was applied to growing maize roots, alterations of the typical distribution patterns of myosin VIII, actin filaments and cortical ER elements associated with plasmodesmata and pit fields were observed [39]. Nevertheless, as shown here, the expressed GFP-ATM1 which is a mutant molecule lacking the head, actin binding domain, did not disrupt the ER network. This suggests that myosin VIII is necessary but not essential for anchoring the cortical ER to the plasma membrane [40] and that other proteins with overlapping functions are there. Since ER spans the plasmodesmata [41] it further suggests close relationships between ATM1 and the ER. Plasmodesmatal myosin VIII was postulated to be involved in regulating conductivity by the force it can generate to control the spacing between desmotubules and plasma membrane [11]. Our data suggest that the cross talk between ER and myosin VIII is not limited to plasmodesmata because colocalization was observed at the outer membrane of epidermal cells. Two models have been proposed for the role of myosin V in ER localization and movement in yeast and animal cells; in model-A Myosin V actively transport ER tubules, while in model-B myosin V plays a role in tethering ER to the cell surface [42]. The prediction is that in plants, myosin XI plays the role of model-A while myosin VIII the role of model-B.

In root hairs (Figure 1D) GFP-ATM1 formed punctuate pattern along the cell. The clear zone of root hairs tip is rich in membrane recycling activity and organelles marked by the endosomal marker FYVE [30]. Myosin XI was also found in the clear zone of pollen tubes [43]. The presence of ATM1 in the clear zone of elongating root hairs should be carefully analyzed.

When ATM1 was co-expressed with different endosomal markers, partial co-localization was found with ARA6, and rarely with ARA7 and FYVE. Endosomes in mammalian cells are categorized into four classes; early endosomes, late endosomes, recycling endosomes and lysosomes [44]. In plants there is no similar classification, and the functional differences between endosomes is not clear. The Rab5 small GTPases typically regulate early endosomes [45] and ARA6 and ARA7, which are plant Rab5 orthologs, localize to different and partially overlapping sub-populations of endosomes [28,46]. While ARA6 co-localized with SNARE proteins characteristic of the pre-vacuolar compartment (PVC), ARA7 did not [46]. This suggested that ARA7 labels an earlier endosomal compartment [46]. Indeed it has been shown that in plants, the routes of endocytosis and vacuolar transport merge at the PVC [47]. However, when ARA7 was co-expressed with other PVC markers – PS1-GFP [48] or AtPEP12p:HA [49], it was also detected in the PVC. In our working system, ATM1 was mainly detected at the plasma membrane in a punctate pattern; however, it was also observed in the cytoplasm, albeit rarely. The dots of cytoplasmic GFP-ATM1 were co-localized with internalized FM4-64 and rarely with the markers FYVE and ARA7. Co-localization of ATM1 with ARA6 was pronounced and seemed to be at the plasma membrane. Indeed it was previously indicated that GFP-ARA6 resides on the plasma membrane and also on the ER [28] whereas Ara7 and Rha1 are different [46]. By showing preference of ATM1 to ARA6 labeled endosomes and by demonstrating that each ARA6 labeled organelle loose its motility when associated with truncated ATM1 while FYVE labeled organelles can remain mobile although associated with ATM1 and only large excess of truncated ATM1 could arrest their motility, we provide another evidence for the presence of different subpopulation of endosomes [46]. Our data suggests that ATM1 is more crucial for the motility of ARA6 containing endosomes and plays only a minor role, if at all, in the function of FYVE/ARA7 containing endosomes. Importantly, the observation that ARA6-GFP partially co-labeled BFA-induced structures in roots [24] is in agreement with our findings of the association of ATM1 with ARA6-GFP on the one hand, and with BFA bodies in specific root cells on the other hand.

The partial localization of ATM1 to different vesicles marked by ARA6, ARA7 or FYVE and to BFA bodies suggests that it has a role in the motility of endosomes at different stages of maturation and in endosomal recycling to the plasma membrane [19,20,22-24]. A dual role in endocytosis, both at the plasma membrane and in endosomal motility, has been suggested for myosin VI [50,51], which is phylogenetically close to myosin VIII [1]. Myosin VI is implicated in both the formation of clathrin-coated vesicles at the plasma membrane and the movement of nascent uncoated vesicles from the actin-rich cell periphery to the early endosome in animal cells [50,51]. Interestingly, myosin VI was found to move toward actin's minus end [52] in a processive manner [53]. The latter functions are still not known for myosin VIII.

### Possible differential roles for myosin VIII in different root cells

Plant roots are very sophisticated sensors that are able to perceive various different environmental and soil cues such as gravitation force, touch, water potential and osmolarity. Gravity-sensing is done by specialized cells that are located within the columella root cap. The signal is perceived by specialized cells, statocytes, containing specific amyloplasts, statoliths that sediment in these cells according to the gravitation force [54]. The gravity perceived signal is transmitted from the columela cells by a mobile auxin signal to the cells at the elongation zone [55]. The actin meshwork is believed to be the cellular structure that sedimenting amyloplasts pass through or interact with to trigger the downstream signaling events leading to root orientation [56]. There is also accumulating evidence that the transport of auxin, is regulated by actin [24,57-59]. Thus the differential localization of ATM1 in root cap and elongating cells might reflect the different roles that it plays as part of the actin cytoskeleton during signaling perception. We did not detected particular phenotypes related to gravitropism in the GFP-ATM1 (IQ-tail) plants. Although ATM1 seems to have polarized localization to transverse walls in cells at the elongation zone its role in the polarized accumulation of auxin transporters [19,24] is still to be determined. It was also shown that touch stimulation but not gravity-stimulation led to transient increases in Ca^2+ ^ions in root cells [60] and that the increase induced in the cap cells was larger and longer-lived than in cells in the meristematic or elongation zone [60]. The touch induced calmodulin like protein 2 (TCH2) [61] was found by us to interact with the IQ domains of ATM1 in a calcium regulated manner [62]. Thus ATM1 can be involved in differential touch responses in the different root cells, being regulated by TCH2 as a light chain. ATM1 was also shown to be responsive to osmo-signals specifically in root-cap cells where it was found to be recruited to plastids surfaces following stimulation [16]. The dotted pattern seen in some of the root cells was identified by an anti myosin VIII antibody in maize roots[11,15] as plasmodesmata enriched pit fields. Using a specific stain for callose we confirmed the presence of ATM1 in pit fields of *N. benthamiana *leaves, however, in the transgenic plants, ATM1 fluorescent dots are scattered all over the cell. Thus we don't know precisely, what is the different function that ATM1 plays in these particular cells. Also, not all cells in the "belt" of cells showing dotted pattern of GFP-ATM1 were dotted, the reason might be the absence of synchronization in their developmental stage.

## Conclusion

While a truncated GFP fusion of ATM1 lacking the head domain can be expressed by plants that remain normal, it was found to be a useful probe for ATM1's behavior in root cells. Using these plants it is shown here, in live cells, that ATM1 changes its localization in root epidermal cells as they develop. This change in localization is accompanied by a change in sensitivity to BFA, indicating on a functional modification. Further this work provide evidence using microscopy of live cells, that Myosin VIII is preferentially involved in the function of ARA6 associated endosomes and is localized to ER and pit fields rich in plasmodesmata.

## Methods

### Plant material

*Arabidopsis thaliana *ecotype Col-0 were seeded on MS (Murashige & Skoog) media, incubated 4 days at 4°C in the dark and then transferred to a growth room at 24°C under 16 h light/8 h dark. After 7–10 days, seedlings were transferred to pots with peat and grown in a temperature-controlled (23°C) greenhouse under continuous light.

*Nicotiana benthamiana *plants were grown in peat in a controlled growth room at 25°C with optimum light for 16 h daily.

### Plasmids

In order to fuse the IQ-tail domain of ATM1 to GFP, we used a cDNA clone kindly provided by Dieter Volkmann (University of Bonn, Germany) and the following primers: Fwd-GGG GTA CCC GTA CTC TCC ACG GCA TT and Rev-CGG GAT CCG TGC TTG GGA ATG CTG CC. The resulting fragment was ligated downstream of GFP using KpnI and BamHI into the plasmid ART7 containing GFP with a linker of 10XAla at its C terminus. Similarly, ATM1 was ligated to ART7 containing RFP cherry [63] with a linker of 10XAla at its C terminus. The ATM2 IQ-tail domain was isolated from Arabidopsis RNA using RT-PCR and the following primers: Fwd-GGG GTA CCA GGA AAA AGG TTC TTC AAG GC and Rev-CGG GAT CCC TAG CCT CTT TTT CCC CA. Similar clones of myosin VIIIA were isolated using these primers: Fwd-GGGGTACCCAGA TTGGGGTTCTTGAAGAT, and Rev-CGGGATCCTTAATACCTAGTA CTCCTCAA. And for myosinVIIIB Fwd-GGGGTACCGTAATTAGCG TCCTTGAGGAA, and Rev-CGGGATCCTCAATAACTTTTCTTGCACCA. These were fused to either GFP or RFP as described for ATM1.

The entire expression cassettes containing the fluorescent chimeras under the regulation of the 35S promoter were then transferred to the binary vector pART27 using a NotI cleavage. The plasmid encoding DsRED-FYVE was provided by Josef Samaj from the University of Bonn [30]. Plasmids encoding GFP fusions of ARA7 and ARA6 were provided by Takashi Ueda from RIKEN, Japan [28]. The plasmid encoding the GFP fusion of the H/KDEL receptor, ERD2-GFP, was provided by Chris Hawes from Oxford Brookes University, UK [26].

### Plant DNA and RNA preparation

DNA was prepared as follows: two to three fresh mature leaves were ground in liquid nitrogen to a fine powder and dissolved in a mixture containing: extraction buffer (350 mM sorbitol, 100 mM Tris PH 7.5, 5 mM EDTA pH 7.5), Nuclei lysis buffer (200 mM Tris PH 7.5, 50 mM EDTA, 2 M NaCl, 2% CTAB) and 5% N-lauroylsarcosine at 1:1:0.4 ratio. After 15 min incubation at 65°C, chloroform/isoamylalcohol extraction was performed. DNA was precipitated in isopropanol and re-suspended in sterile ddH2O. Total RNA was prepared as follows: 2.5 gr of plant tissue was ground in liquid nitrogen to a fine powder and incubated 10 min in 20 ml hot (65°C) CTAB buffer (2% PVP, 2% CTAB, 2 M NaCl, 25 mM EDTA PH8, 0.1 M Tris PH8). Chloroform/isoamylalcohol extraction was followed and RNA was precipitated in 2.5 M LiCl. The pellet was re-dissolved in SSTE buffer (1 mM EDTA, 10 mM Tris PH8 1 M NaCl, 0.5% SDS) and additional chloroform/isoamylalcohol extraction was performed. RNA was precipitated in ethanol and NH_4_Ac and the pellet was dissolved in sterile double distilled ddH_2_O. Poly-A RNA was isolated using Oligotex kit of Qiagen (Cat No. 7002) according to the manufacturer's instructions. RT-PCR was performed using the SuperScript reverse transcriptase of Invitrogen (Cat No. 18064-022).

### Western blot analysis

The amount of 250 mg of seedlings of transgenic plants was grinded to a fine powder in liquid nitrogen. The powder was boild in 200 μl of Laemmli's protein sample buffer [64] for 10 min and then centrifuged 10 min at 14 krpm in room temp. A sample (30 μl) of the extract was separated on SDS-PAGE and blotted onto a nitrocellulose membrane. GFP fusion proteins were detected with anti GFP antibody (Santa Cruz), and a secondary HRP conjugated antibody (Jacksom ImmunoResearch). For chemiluminescent reaction, the SuperSignal kit (Pierce) was used.

### Fluorescent microscopy and staining

An IX81/FV500 laser-scanning microscope (Olympus) was used to observe fluorescently labeled cells. The following filter sets were used: for observing GFP, 488 nm excitation and BA505-525; RFP, 543 nm excitation and BA610; FM4-64, 488 or 515 nm excitation and BA660. The objective used was PlanApo 60 × 1.00 WLSM 8/0.17. To observe aniline blue we used 405 nm excitation and BA430-460, using objective UPlanSApo 60 × 1.35 oil, 8/0.17 FN26.5. When GFP and RFP were detected in the same sample, we used DM (dichroic mirror) 488/543 and when aniline blue was added, DM 405/488/543/633 was used. In all cases, where more than one color was monitored, sequential acquisition was performed. FM4-64 (Molecular Probes) staining was performed at a final concentration of 8 μM for 5–20 min. Callose was stained by incubating leaf segments for 40 min in a mixture of 0.1% aniline blue in ddH_2_O and 1 M glycine, pH 9.5, at a volumetric ratio of 2:3, and pre-mixed for at least 1 d before use [27]. BFA (Sigma) was used at a final concentration of 50 μM for 50 min. in the presence of 4 μM of FM4-64.

### Agrobacterium infiltration into *N. benthamiana *leaves

The fluorescent chimeras were expressed using Agrobacterium infiltration. Briefly: *Agrobacterium tumefaciens *strain GV3101 was transformed with the plasmid and grown at 28°C for 48 h. Bacterial culture originating from one colony was grown, precipitated and dissolved to an OD_600 _of 0.5 in the following buffer: 50 mM MES, pH 5.6, 0.5% glucose, 2 mM NaPO_4 _and 100 μM acetosyringone (cat. no. D13440-6, Sigma Aldrich). Leaves of 3-week-old *N. benthamiana *plants were infiltrated with the bacterial culture using a 1-ml syringe. Expression of the fluorescent chimera in the leaf cells was detectable after 24 h, peaking at 48 h.

### Transgenic Arabidopsis plants

Arabidopsis plants were transformed as previously described [65]. A culture of Agrobacterium GV3101 was grown in 1 liter of LB for 24 h at 28°C. The bacteria were precipitated at 5000 rpm for 15 min and dissolved to an OD_600 _of 0.8 in the following buffer: 5% sucrose, 10 mM MgCl_2_, and 0.044 μM BA (benzylamino purine). The detergent Silwet L-77 (LHELE SEEDS Texas USA) was added to a final concentration of 0.03%. Plants at the closed-flower-bud stage were dipped in the Agrobacterium solution for 5 min. The plants were then covered with plastic bags and transferred to controlled growth conditions. After 48 h, the bags were removed and after 1 month, seeds were collected. Seeds were sterilized in 1% NaClO for 5 min and washed three times with sterile H_2_O. Seeds (~5000) were plated on 10-cm Petri dishes with MS, the relevant antibiotics and 500 μg/ml claforan (Aventis) to prevent Agrobacterium growth. Antibiotic-resistant seedlings were transferred to a new Petri dish and scanned for GFP or RFP expression under a fluorescent binocular.

## Authors' contributions

LG performed the cloning, transient expression, transgenic plants and microscopy, MA helped with cloning and transgenic plants and EB with microscopy. ES coordinated the study and wrote the manuscript.

## Supplementary Material

Additional file 1 Three dimension reconstruction of lateral root of GFP-ATM1(IQ-tail) expressing plant. Rotation of the 3D image shows that the ATM1-dots are scattered in the cells at different optic planes. Click here for file

Additional file 2 **BFA and FM4-64 treatment to GFP expressing seedlings**. Five day old seedlings expressing GFP were subjected to BFA and FM4-64 treatment as described for ATM1 expressing plants. A. GFP B. FM4-64. C. overlay of A and B. Scale bar 5 μm, 1 optic section. Click here for file

Additional file 3 Time lapse movie of RFP-ATM1 (IQ-tail) expressed in an abaxial leaf epidermal cell of *N. benthamiana*. Images were acquired every 1.3 second and are run at 8 frames per second. It is shown that ATM1 dots are mostly stationary but rarely motile dots are seen. Click here for file

Additional file 4 Expression profiles of the four members of myosin VIII in the different plant organs as determined by Genevestigator. Click here for file

Additional file 5 Time lapse movie of GFP-ARA7 and RFP-ATM1(IQ-tail) expressed in an abaxial leaf epidermal cell of *N. benthamiana*. Images were acquired every 1.3 second and are run at 8 frames per second. It is shown that while GFP-ARA7 labeled organelles are moving in the cytoplasm, ATM1 dots are stationary at the plasma membrane. The difference between the plasma membrane depicted by the ATM1 labeling and the rapidly moving cytoplasm and organelles is emphasized in this movie. Scale bar 5 μm. Click here for file

Additional file 6 Time lapse movie of GFP-ATM1(IQ-tail) and DsRed-FYVE expressed in an abaxial leaf epidermal cell of *N. benthamiana*. Images were acquired every 1.3 second and are run at 8 frames per second. Motile FYVE labeled organelles are shown, some of them associated with ATM1. The speed of movement is sometime faster than the time of switch between the two lasers. This is seen by the separation of the green and magenta dots in some of the frames. Scale bar 5 μm. Click here for file

Additional file 7 Time lapse movie of GFP-ATM1(IQ-tail) and DsRed-FYVE expressed in an abaxial leaf epidermal cell of *N. benthamiana*. Images were acquired every 1.3 second and are run at 8 frames per second. Motile FYVE labeled organelles are shown, but one of them which is completely surrounded by GFP-ATM1 is stationary. Click here for file

Additional file 8 Time lapse movie of ARA6-GFP expressed in an abaxial leaf epidermal cell of *N. benthamiana*. Images were acquired every 1.3 second and are run at 8 frames per second. Motile ARA6-GFP labeled organelles are shown. Click here for file

Additional file 9 Time lapse movie of ARA6-GFP and RFP-ATM1(IQ-tail) expressed in an abaxial leaf epidermal cell of *N. benthamiana*. Images were acquired every 1.3 second and are run at 8 frames per second. It is shown that only ARA6-GFP organelles free from RFP-ATM1 are motile while all the double labeled organelles are motionless. Scale bar 5 μm. Click here for file
