# Supplementary figures and images for: Different subcellular localizations and functions of Arabidopsis myosin VIII
Source: BMC Plant Biol. 2008 Jan 8;8:3. doi: 10.1186/1471-2229-8-3 (PMC2275265; doi:10.1186/1471-2229-8-3)

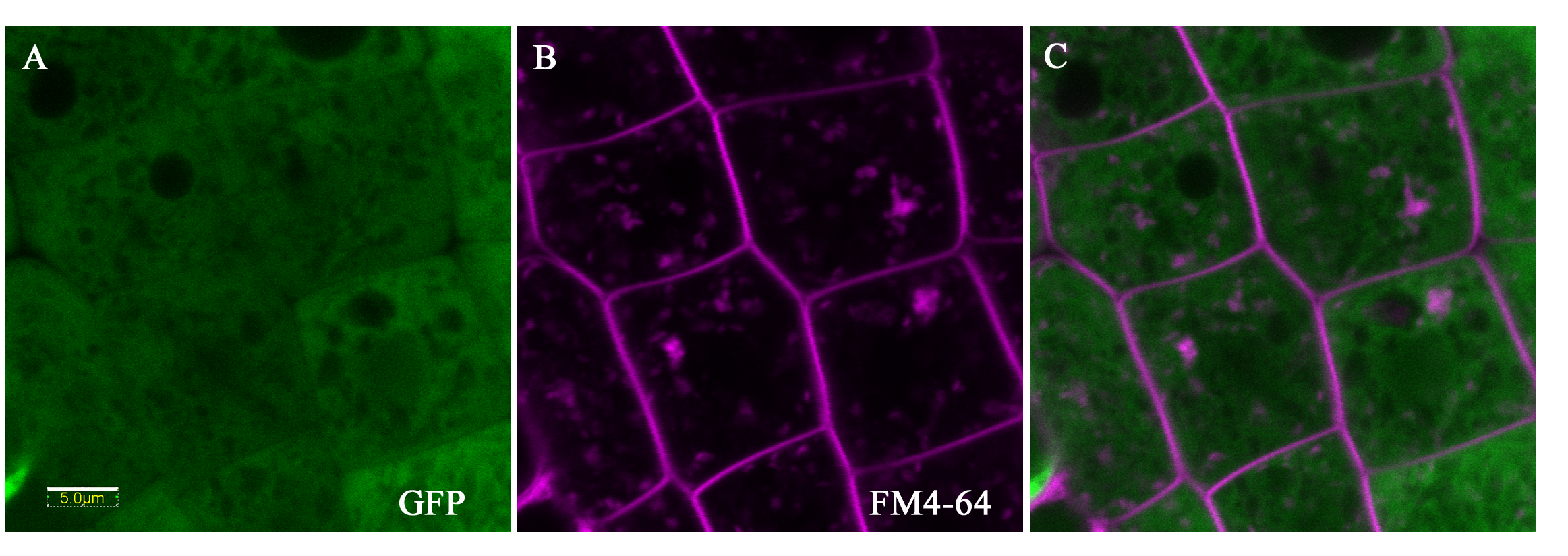

Supplement: Additional file 2 — BFA and FM4-64 treatment to GFP expressing seedlings. Five day old seedlings expressing GFP were subjected to BFA and FM4-64 treatment as described for ATM1 expressing plants. A. GFP B. FM4-64. C. overlay of A and B. Scale bar 5 μm, 1 optic section. [file 1471-2229-8-3-S2.TIFF]
